# *In Silico* Identification and Evaluation of Leads for the Simultaneous Inhibition of Protease and Helicase Activities of HCV NS3/4A Protease Using Complex Based Pharmacophore Mapping and Virtual Screening

**DOI:** 10.1371/journal.pone.0089109

**Published:** 2014-02-13

**Authors:** Abdul Wadood, Muhammad Riaz, Reaz Uddin, Zaheer ul-Haq

**Affiliations:** 1 Computational Medicinal Chemistry Laboratory, Department of Biochemistry Abdul Wali Khan University Mardan, Mardan, Khyber Pakhthunkhwa, Pakistan; 2 Dr. Panjwani Center for Molecular Medicine and Drug Research, University of Karachi, Karach, Sindh, Pakistan; Wake Forest University, United States of America

## Abstract

Hepatitis C virus (HCV) infection is an alarming and growing threat to public health. The present treatment gives limited efficacy and is poorly tolerated, recommending the urgent medical demand for novel therapeutics. NS3/4A protease is a significant emerging target for the treatment of HCV infection. This work reports the complex-based pharmacophore modeling to find out the important pharmacophoric features essential for the inhibition of both protease and helicase activity of NS3/4A protein of HCV. A seven featured pharmacophore model of HCV NS3/4A protease was developed from the crystal structure of NS3/4A protease in complex with a macrocyclic inhibitor interacting with both protease and helicase sites residues via MOE pharmacophore constructing tool. It consists of four hydrogen bond acceptors (Acc), one hydrophobic (Hyd), one for lone pair or active hydrogen (Atom L) and a heavy atom feature (Atom Q). The generated pharmacophore model was validated by a test database of seventy known inhibitors containing 55 active and 15 inactive/least active compounds. The validated pharmacophore model was used to virtually screen the ChemBridge database. As a result of screening 1009 hits were retrieved and were subjected to filtering by Lipinski’s rule of five on the basis of which 786 hits were selected for further assessment using molecular docking studies. Finally, 15 hits of different scaffolds having interactions with important active site residues were predicted as lead candidates. These candidates having unique scaffolds have a strong likelihood to act as further starting points in the development of novel and potent NS3/4A protease inhibitors.

## Introduction

HCV infection has been declared as a principal health problem in more than 200 million individuals throughout the world [1]. It is a positive-stranded RNA virus and classified as a hepacivirus of the flaviviridae family [2]. Unlike other viral infections Hepatitis C Virus even with its high replication rate can stick within a human host for decades without any irritation or liver damage [3]. Estimated 10 million people are believed to be infected by HCV alone in Pakistan [4]. Eventually the infection causes severe complications in 60 to 70% of patients such as cirrhosis, fibrosis, liver failure and hepatocellular carcinoma [5]. Prior to the development of HCV protease inhibitors combination therapy, patients with HCV infection were treated with pegylated interferon-α and ribavirin [6]. The adverse side effects associated with this type of treatment such as anemia, flu-like symptoms, depression, gastrointestinal symptoms, fatigue and cutaneous reactions may lead to the discontinuation of treatment in certain number of patients [7]. Moreover, this treatment was found successful only in 50% people with genotype 1 infection [8], [9]. The considerable side effects, lower competence of this treatment and more commonness of the infection throughout the world demanded for more efficient and sound-tolerated medication [10], [11]. The growth in scientific knowledge of HCV life cycle and its replication leads to the development of inhibitors of HCV proteases [12], [13].

**Figure 1:**
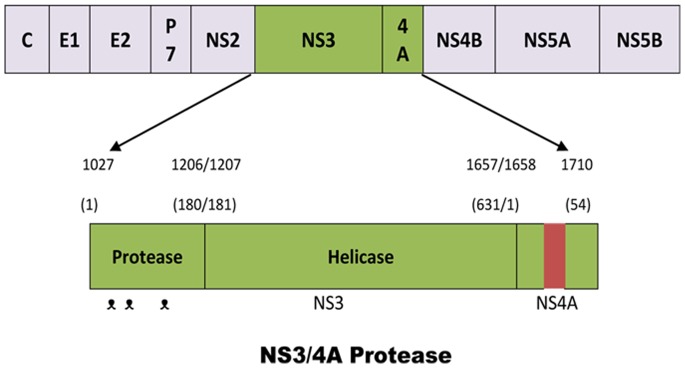
Schematic representation of the HCV NS3/4A protease. The amino acid position for the domain and sub-domain is indicated as a number either starting from the 1st amino acid of the entire polyprotein (the number at the top) or starting from the 1st amino acid of the NS3 or NS4A (the number at the bottom). On the NS3/4A protease, the catalytic triad, namely His-1083, Asp-1107 and Ser-1165 of the polyprotein (or His-57, Asp-81 and Ser-139 of the NS3), is indicated as “
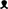
”. The reddish box in the NS4A indicates the 14-amino acid central hydrophobic region of NS4A (amino acids 1678–1691 of the polyprotein or amino acids 21–34 of the NS4A), which has been shown to be sufficient for activation of the NS3 protease activity [17].

A polyprotein precursor encoded by HCV RNA genome containing structural proteins capsid (C), membrane (prM), envelope (E) and nonstructural (NS) proteins (NS1, NS2a, NS2b, NS3, NS4a, NS4b, NS5) [14]. NS3 protease when activated by NS4A causes the cleavage of polyprotein producing the non-structural proteins 4A, 4B, 5A, 5B and is thus very supportive in the replication of virus [15], [16]. That is why; NS3/4A protease is a significant emerging target for the treatment of HCV infection. The full-grown NS3 protein contains the amino acids ranging from 1027 to 1657 of the HCV polyprotein [17]. NS3 protease consists of an N-terminal protease domain and a C-terminal helicase domain [18]. The N terminal 180 amino acids of NS3, ranging from 1027 to 1206 contains the protease activity and the remaining 450 amino acids i.e. from 1207 to 1657 are associated with helicase activity [19]–[20] (Fig. 1). The protease and helicase domains of NS3 protease have their individual functions i.e. NS3/4A protease causes polyprotein processing and helicase activity is RNA replication [21]. In addition, it has also been found that protease increases the helicase activity and the protease activity is enhanced by the helicase [22]. The active site configuration of NS3 protease comprises the residues His-57 (His-1083), Asp-81 (Asp-1107), and Ser-139 (Ser-1165) [23](Fig. 1). NS3 protease requires the vital 14-monomer hydrophobic peptide NS4A for its activation [24]. A stable complex between NS3 and NS4A is formed on the endoplasmic reticulum (ER) membrane in transfected cells [15]. About 30 amino acids at the N terminus of NS3 interact with NS4A for complex formation [25]. NS4A shows a dual role in complex, first the proteolytic activity of NS3 is enhanced by it and secondly it targets the NS3 protein to the ER membrane i.e. NS3 associates to the ER membrane only in the presence of NS4A [25]–[26].

The previous studies show that the NS3 protease is structurally similar to the trypsin family but features like a deep active site, a structural non catalytic zinc ion, and dependence on another viral cofactor NS4A, make it distinctive [17]. Such features imposed a big challenge in developing small and potent inhibitors of the NS3/4A protease. Currently, the inhibitors of NS3/4A were found promising candidates for the treatment of HCV infection [27], [28]. There are several inhibitors of HCV NS3/4A protease that are passing through clinical improvement showing good potency against HCV infection in a number of patients [29]. However, a rapid viral resistance was observed to be developed in patients treated with such inhibitors [30]. Therefore, continuous efforts are desirable for the development of more potent NS3 protease inhibitors that have different resistance profile and binding mode.

The development of computational methods and the wide applications of *in silico* screening were stimulated by the low hit rates and high cost associated with wet lab. Recently, in drug discovery process the high-throughput virtual screening is becoming complementary to high-throughput screening in an effort to develop new and potent drug like molecules [31]. Hence, to identify novel and potent ligands which inhibit both the protease and helicase activity of NS3/4A protein of HCV, a complex-based pharmacophore modeling and virtual screening might be considered as an efficient approach. A pharmacophore comprises the 3D arrangements of chemical or structural features of small organic compounds used as drug that may be important for interaction with the target. These pharmacophores can be used in drug design programs in different ways: (1) as 3D query in *in silico* screening to identify new and potent hits with the structures different from the already reported poses from the databases of “drug-like” molecules; (2) to predict the activities of novel compounds before synthesis; (3) to know the probablemode of action [32], [33].

This work reports the complex-based pharmacophore modeling to find out the important pharmacophoric features essential for the inhibition of both protease and helicase activity of NS3/4A protein of HCV by virtual screening, drug-likeness predictions, molecular docking, protein-ligands binding interactions, binding affinity predictions and binding energy calculations. The identification of fifteen novel and potent lead compounds as NS3/4A protease inhibitors clearly reflects the significance of this study.

## Materials and Methods

### Generation and Validation of Complex-Based Pharmacophore Model

**Figure 2:**
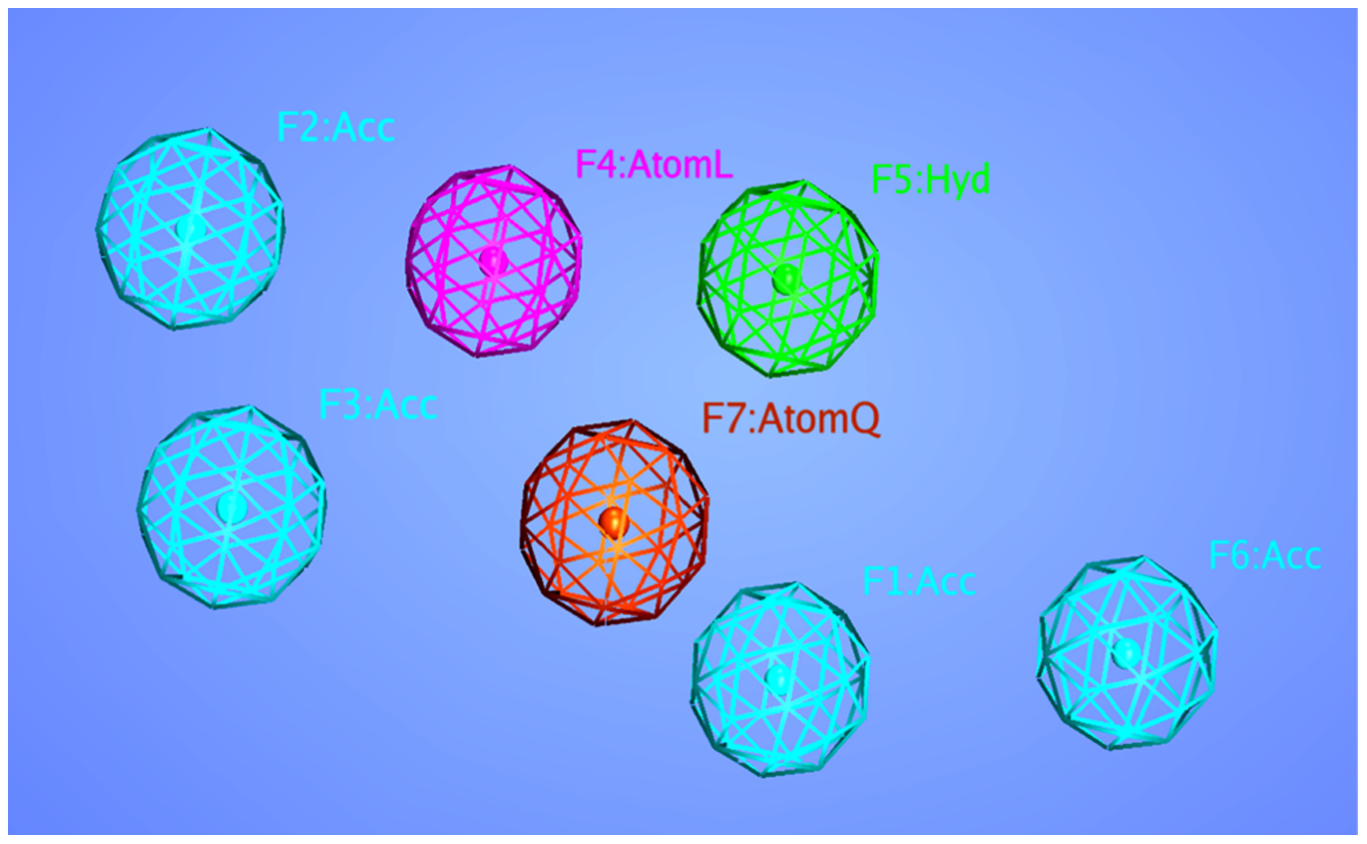
Three-dimensional pharmacophoric features generated from complex structure of HCV NS3/4A protease.

Pharmacophore is an ensemble of steric and electronic features that is necessary to ensure the optimal supramolecular interactions with a specific biological target and to block its biological response [34]. The complex-based pharmacophore technique can be used to advance the drug development process if the 3D structure of the target protein is available. In this study the crystal structure of NS3/4A protease in complex with a macrocyclic inhibitor interacting with both protease and helicase active sites residues (4a92) [18] was used for the generation of complex-based pharmacophore model. Pharmacophore constructing tool implemented in MOE (Molecular Operating Environment) was used for the generation and visualization of 3D pharmacophore from structural data of protein-ligand complex. The important chemical features for the generation of pharmacophore model were identified on the basis of binding interactions observed in the protein-ligand complex. A total of seven key features including four hydrogen bond acceptors (Acc), one hydrophobic (Hyd), one for lone pair or active hydrogen (Atom L) and a heavy atom feature (Atom Q) were created in the resulting pharmacophore model using the default parameters of MOE. Among these seven features, four features (F1, F2, F3, F4), were marked as essential features (Fig. 2). The generated pharmacophore model was validated by a test database of seventy known inhibitors of HCV NS3/4A protease. All compounds of the test database were screened on the seven-featured complex-based pharmacophore and their mapping modes were analyzed.

### Pharmacophore-Based Database Screening

The validated pharmacophore model was used as 3D query in *in silico* screening to identify hits of various chemical natures. Using the software MOE, pharmacophore based virtual screening was carried out against already prepared ChemBridge database [35]. Such type of virtual screening has two main purposes: first, the quality of generated pharmacophore model is validated by selective detection of compounds with known inhibitory activity, and second, identifying novel, potent drug like poses for further evaluation [36]. As a result of screening, 1009 structurally diverse hits were retrieved from ChemBridge database exhibiting a better six features fit to the generated pharmacophore model. In order to know whether the retrieved hits possess drug-like properties, the properties of each hit ligand were studied for Lipinski’s rule of five. Strictly following the Lipinski’s rule of five, finally, 786 hits were selected for further assessment.

### Molecular Docking

To further refine the hit compounds, all the retrieved hits were docked into the binding site of NS3/4A protease. A number of docking programs are available for molecular docking studies. Here, we used Docking protocol implemented in MOE as a docking program [37]. The crystal structure of NS3/4A protease complex with a macrocyclic inhibitor was obtained from protein data bank and was prepared for docking as described in our previous work [38]. A maximum of 10 conformations were allowed to be saved for each ligand using the default parameters of MOE (Placement: Triangle Matcher, Rescoring 1: London dG, Refinement: Forcefield, Rescoring 2: GBVI/WSA dG). The top ranked conformations of all docked compounds were saved in a separate database. On the basis of docking score, 300 top ranked compounds were selected for further evaluation. The ligands were ranked by the scores from the Generalized-Born Volume Integral/Weighted Surface Area (GBVI/WSA) binding free energy calculation in the **S** field which is the score of the last stage. The GBVI/WSA is a scoring function which estimates the free energy of binding of the ligand from a given pose. For all scoring functions, lower scores indicate more favorable poses. The resulted binding interactions between these 300hits and protein were observed using LigPlot implemented in MOE.

### Binding Energy and Binding Affinity Calculations

To identify the most potential ligands, binding affinities of the hits-NS3/4A protease complexes were calculated with generalized Born / volume integral (GB / VI) implicit solvent method implemented in MOE [39]. Generalized Born interaction energy is the non-bonded interaction energy between the receptor molecule and the ligand that includes Coulomb electrostatic interaction, Vander Waals, and implicit solvent interaction energies. The strain energies of ligands and receptor molecules are, however, not taken into account. During calculation solvent molecules were ignored. The estimated binding affinity is that of the *London dG* scoring function reported in unit of Kcal/Mol. During calculations the atoms of the receptor molecule away from the ligand were kept rigid while receptor atoms in the locality of the ligand (in the binding site) were kept flexible but were subjected to tether restraints that discourage gross movement. The ligand atoms were set free to move at the binding pocket. In each case an energy minimization of binding pocket inNS3/4A protease–ligand complex was performed before calculating binding affinity. The binding affinity was calculated for each hit after energy minimization, and reported in unit of Kcal/Mol.

## Results and Discussion

### Generation and Validation of Complex-Based Pharmacophore Model

**Figure 3:**
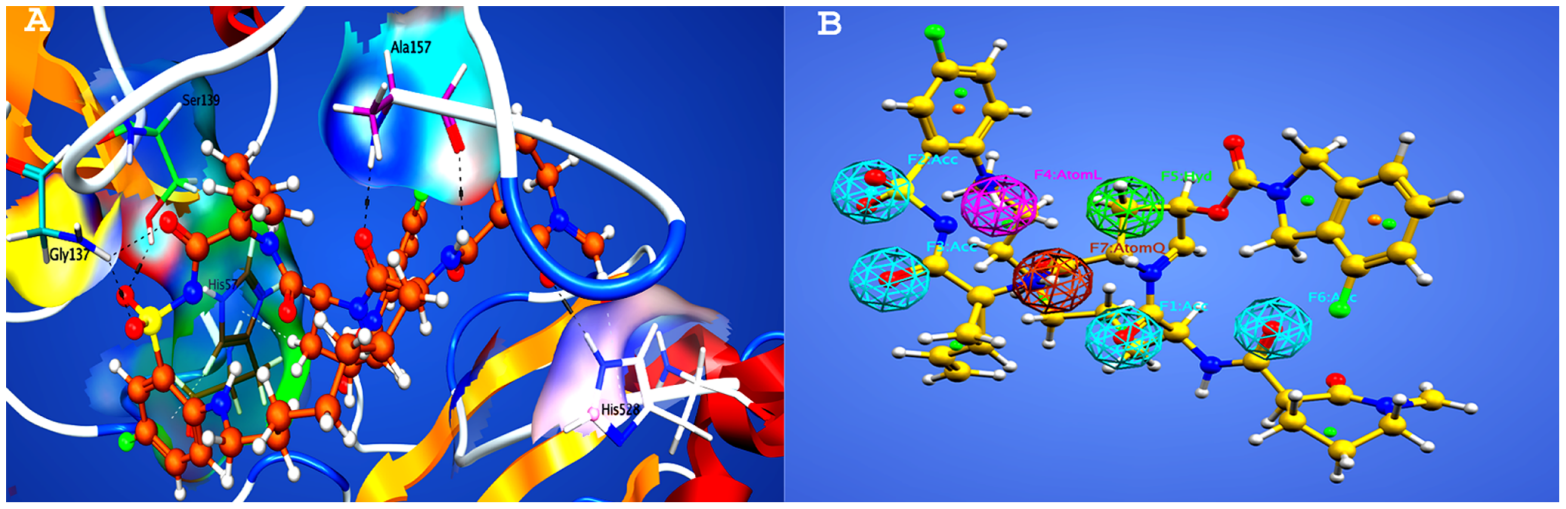
Complex structure of reference ligand and NS3/4A protease. (A) Three-dimensional representation of the interactions of reference ligand and receptor protein NS3/4A protease. (B) Three-dimensional pharmacophore model generated from complex structure of HCV NS3/4A protease. Cyan, brown, purple and green colors represent hydrogen bond acceptor, heavy atom, lone pair or active hydrogen and hydrophobic features respectively.

An interesting application of complex-based pharmacophore model is to determine interaction points so as to lead the improvement of binding affinity and increasing selectivity. A complex-based pharmacophore model was generated from the crystal structure of NS3/4A protease in complex with a macrocyclic inhibitor using pharmacophore constructing tool implemented in MOE. On the basis of binding interactions, observed in the protein-ligand complex via LigPlot implemented in MOE, the important chemical features for the generation of pharmacophore model were identified. A total of seven key features including four hydrogen bond acceptors (Acc), one hydrophobic (Hyd), one for lone pair or active hydrogen (Atom L) and a heavy atom feature (Atom Q) were created in the resulting pharmacophore model using the default parameters of MOE. Cyan, brown, purple and green colors are represented by hydrogen bond acceptor, heavy atom, lone pair or active hydrogen and hydrophobic features respectively (Fig. 3). The hydrogen bond acceptors features (Acc) were developed on the oxygen atoms of sulfonamide group and on the three carbonyl oxygen of the ligand owing to their binding interactions with important active site residues, Ser 139, Gly 137, Ala 157 and His 528. The hydrophobic (Hyd) feature locates the atom involved in interaction with His 57, the active site residue. To increase the specificity of generated pharmacophore model the two other features, Atom L and Atom Q were also created. Among these seven features, four features (F1, F2, F3, F4), were marked as essential features. The generated pharmacophore model was validated by a test database of seventy known inhibitors containing 55 active and 15 inactive/least active compounds. These seventy inhibitors of the test database and their inhibitory activities were collected from the previous literature [40]–[44]. The 3D structures of the test set compounds were constructed using MOE builder tool and were energy minimized via MOE energy minimization algorithm [Gradient: 0.05, Force Field: MMFF94X]. All compounds of test database were screened on the seven-feature complex-based pharmacophore and their mapping modes were analyzed. Interestingly, 54 out of 55 active compounds, with their 90 different conformations, were obtained as hits mapping six features of the generated pharmacophore model. It is also attractive to note that none of the inactive/least active compound was mapped to seven-feature complex-based pharmacophore model. These results of the test database reflect the accuracy of our developed pharmacophore model.

### Pharmacophore-Based Database Screening

The validated pharmacophore model was used to screen compounds with similar features from the ChemBridge database to find other novel structural poses that fulfill the specified criteria of the model. This is a useful method that can be used for fast finding of novel, potent, drug like compounds in a medicinal chemistry research project. As a result of screening, 1009 structurally diverse hits were retrieved from ChemBridge database exhibiting a healthier six feature fit to the generated pharmacophore model. In order to know the druggability of retrieved hits, the properties of each hit ligand were checked for Lipinski’s rule of five. Lipinski’s “rule of five” states that drug like molecules should contain molecular weight less than 500 *Da*, *log P* value less than 5, hydrogen bond donors less than 5 and hydrogen bond acceptors less than 10 otherwise they have poor permeation or absorption [45]. Strictly following the Lipinski’s rule of five, finally, 786 hits were selected for further assessment using molecular docking.

### Molecular Docking

**Figure 4:**
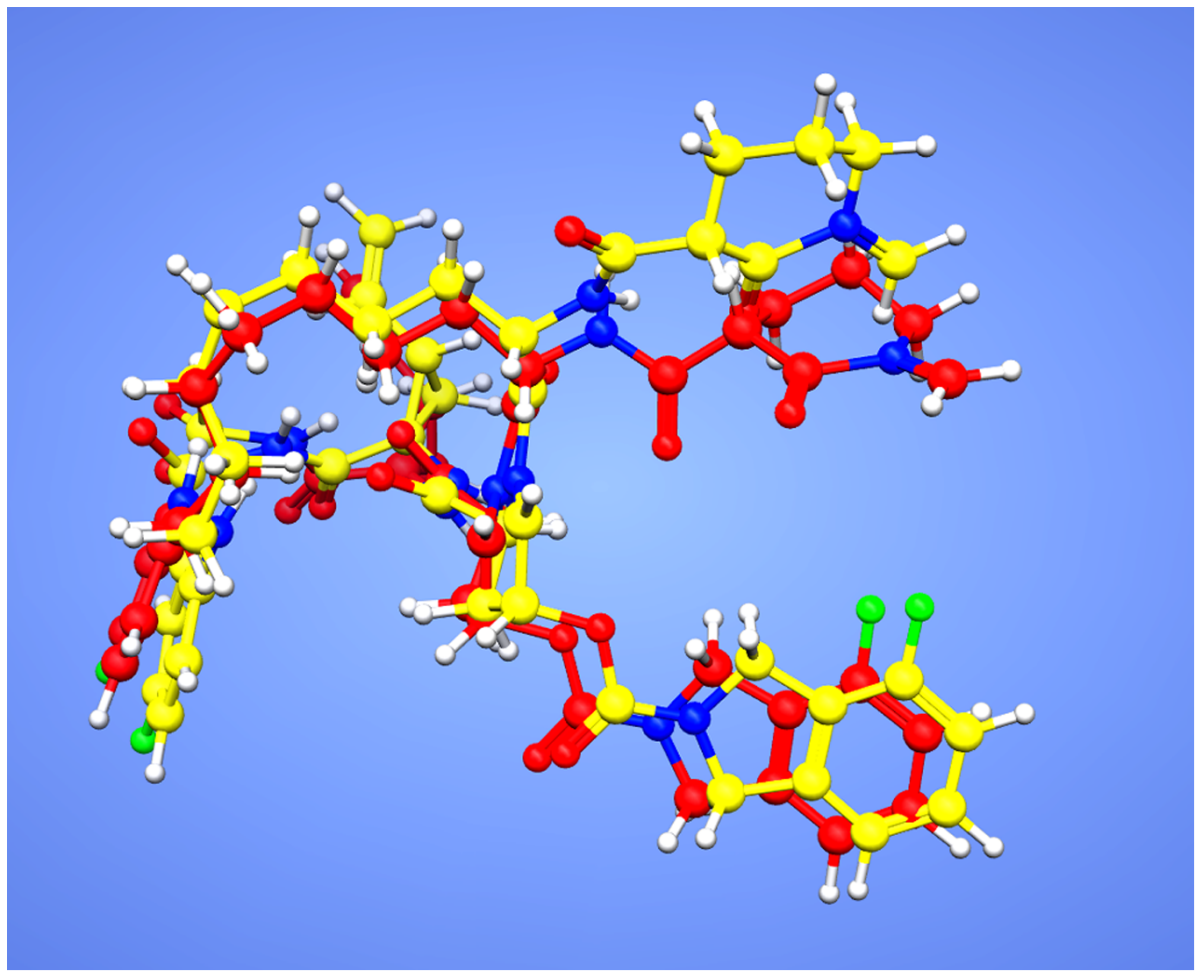
Superposition of co-crystallized and docked conformations of the reference ligand. Red Native co-crystallized ligand and yellow docked ligand.

To further refine the hit compounds, all the initially retrieved hits were docked into the binding site of NS3/4A protease using the docking protocol implemented in MOE. Before docking the initial hits, the ligand from the complex structure was extracted and re-docked into the binding cavity of protein to validate the docking protocol. The root mean square deviation (RMSD) between the co-crystallized and re-docked conformation was calculated by using SVL script of MOE and found to be equal to 1.83 Å (Fig. 4), suggesting that our docking protocol is reliable in reproducing the experimentally determined binding mode for corresponding protein-ligand complex. The MOE docking protocol and the parameters set could be used to search the binding modes of other compounds accordingly. Using the same docking protocol all the initial hits were docked into the binding pocket of HCV NS3/4A protease. A maximum of 10 conformations were allowed to be saved for each ligand using the default parameters of MOE already discussed. The top ranked conformations of all docked compounds were saved in a separate database. On the basis of docking score, 300 top ranked compounds were selected for further evaluation. The resulted binding interactions between these 300 hits and protein were visually observed using LigPlot implemented in MOE and those molecules which revealed significant interactions with most of the important binding pocket residues (His 57, Lys 136, Ser 139, Gly 137,Arg 155,Ala 157, Ala 156 of protease site and Met 485, Glu526, His 528 of helicase site) of HCV NS3/4A protease were selected as promising hits. Among these 300 compounds, 52 showed crucial interactions with the important residues of target protein. These 52 compounds were further subjected to Binding energy and Binding affinity calculation.

### Binding Energy and Binding Affinity Calculations

**Figure 5:**
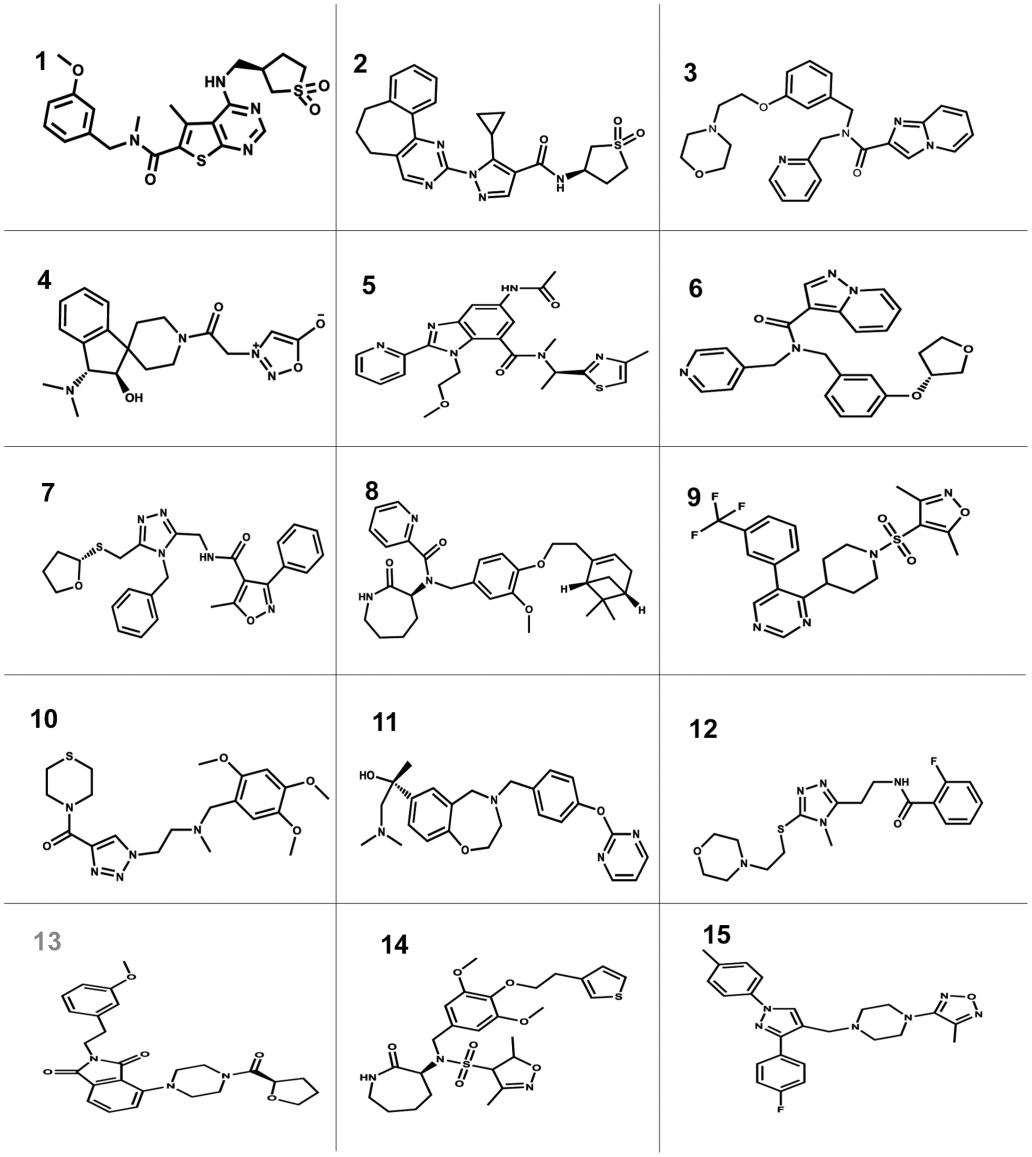
2D structures of retrieved hits from ChemBridge database.

To identify the most potential ligands, binding affinities for all the 52 compounds including ligand of the complex structure were calculated with generalized Born/volume integral (GB/VI) implemented in MOE. In each case an energy minimization of binding pocket in NS3/4A protease–ligand complex was performed before calculating binding affinity. The binding affinity was calculated for each hit after energy minimization, and reported in unit ofKcal/Mol. The selection criteria for the most promising candidates were, compounds having binding energy and binding affinity good or equal to that calculated for the reference ligand in the complex structure, visualization of each hit in the binding cavity and the selection of only those hits showing interactions with important residues in binding cavity of HCV NS3/4A protease. Applying the above mentioned criteria, out of 52, only 15compounds fulfill the particular requirements (Table 1). The pharmacophore mapping, binding mode, binding affinity, binding energy and visual prediction showed that these predicted lead compounds might act as novel, potent and structurally diverse inhibitors of HCV NS3/4A protease. The 2D structures of these retrieved hits are shown in Fig. 5.

**Table 1:** ChemBridge database ID, Docking Scores, binding energies, binding affinities and drug like properties of hit compounds.

Compound	ChemBridgeID	DockingScore (S)	Binding affinityKcal/mol	Binding energyKcal/mol	Drug like properties
1	74212070	−12.1129	−6.40	−23.04	MW.474.61 g/mol, LogP. 3.39, LogS. −5.37, Don. 1, Acc. 6
2	13203524	−11.4164	−6.17	−23.49	MW. 463.56 g/mol, LogP. 2.61, LogS. −5.71, Don. 1, Acc. 6
3	20259391	−11.1719	−6.71	−28.47	MW. 471.56 g/mol, LogP. 3.86, LogS. −3.48, Don. 0, Acc. 6
4	27798935	−11.0684	−5.92	−19.83	MW. 372.42 g/mol, LogP. 1.01, LogS. −2.12, Don. 1, Acc. 5
5	92175699	−11.0708	−7.38	−25.09	MW. 492.60 g/mol, LogP. 4.66, LogS. −4.60, Don. 1. Acc. 6
6	63465583	−10.9940	−5.99	−18.69	MW. 429.48 g/mol, LogP. 4.01, LogS. −3.47, Don. 0, Acc. 6
7	60321457	−10.9496	−7.87	−27.87	MW. 489.60 g/mol, LogP. 5.02, LogS. −7.06, Don. 1, Acc. 5
8	93854211	−10.8731	−7.98	−31.22	MW. 517.67 g/mol, LogP. 5.43, LogS. −6.53, Don. 1, Acc. 5
9	34215248	−10.8285	−6.46	−23.60	MW. 466.48 g/mol, LogP. 4.65, LogS. −5.37, Don. 0, Acc. 5
10	97464457	−10.6463	−7.37	−27.11	MW. 435.55 g/mol, LogP. 2.16, LogS. −2.39, Don. 0, Acc. 7
11	10355774	−10.6286	−7.54	−31.77	MW. 434.54 g/mol, LogP. 4.28, LogS. −4.51, Don. 1, Acc. 6
12	45481066	−10.6055	−7.52	−25.09	MW. 393.49 g/mol, LogP. 1.71, LogS. −3.79, Don. 1, Acc. 5
13	51314220	−10.6047	−7.70	−26.07	MW. 463.53 g/mol, LogP. 2.36, LogS. −4.65, Don. 0, Acc. 5
14	35611883	−10.5838	−7.25	−29.84	MW. 563.70 g/mol, LogP. 4.12, LogS. −4.90, Don. 1, Acc. 7
15	37363620	−10.4503	−6.48	−19.49	MW. 432.50 g/mol, LogP. 4.27, LogS. −5.71, Don. 0, Acc. 4
16	Reference	−12.789	−11.25	−41.70	MW. 865.96 g/mol, LogP. 3.17, LogS. −8.61, Don. 4, Acc.8

### Binding Interactions of Finally Selected Compounds

**Figure 6:**
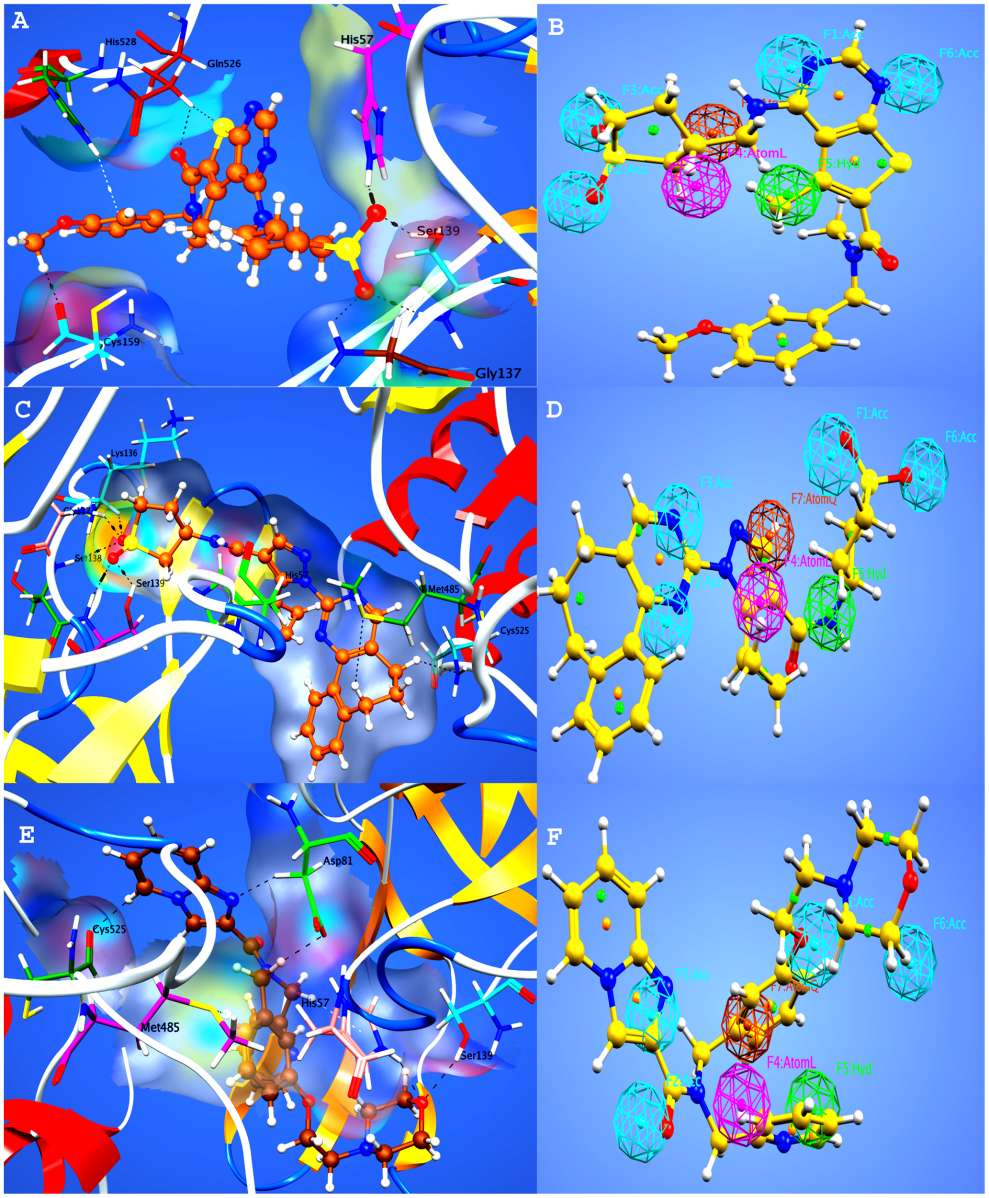
Docking conformations and pharmacophore mapping of compounds 1, 2 and 3. (A) Three-dimensional representation of the interactions of compound 1 and target protein. (B) Pharmacophore mapping of hit compound 1. (C) Three-dimensional representation of the interactions of compound 2 and target protein. (D) Pharmacophore mapping of hit compound 2. (E) Three-dimensional representation of the interactions of compound 3 and target protein. (F) Pharmacophore mapping of hit compound 3.

It was observed in the docking studies that all finally selected hits showed significant binding interactions with the important residues of protease as well as helicase site of the target protein. For example, compound **1**, for which the strong binding affinity (−6.40 Kcal/Mol), lower binding energy (−23.04 Kcal/Mol) and good docking score (−12.1129)was observed, showed the binding interaction with the protease and helicase binding site residues (Fig. 6A). From the top-ranked docked conformation, it was also observed that a phenyl ring, thienopyrimidine moiety and an adjacent carbonyl oxygen of the compound **1** establishedinteractions with the residues of helicase binding site of the target protein whereas a terminal methoxy group and two oxygen atoms of thiophene interact with the protease binding site residues. The helicase binding site residues, His 528 made an arene-hydrogen interaction with the phenyl ring and Gln 526 made two polar interactions with the carbonyl oxygen and thienopyrimidine moiety. From the protease site, the residues, His 57, Ser 139, and Gly 137 are involved in hydrogen bonding with oxygen atoms of thiophenering and Cys 159 with the methoxy group of compound. The pharmacophore mapping of compound **1** is shown in Fig. 6B.

The top-ranked docking pose of compound **2** showed that hydrogen atoms of cycloheptane group of the compound showed interactions to Met 485 and Cys 525 of helicase site residues of the enzyme (Fig. 6C). The protease site residues, Lys 136, Gly 137, Ser 138 and Ser 139 all showed hydrogen bonding with the oxygen atoms of terminal thiophene ring of compound. Moreover, His 57 was also found within a distance of π-π interaction with pyrazole moiety of compound. The pharmacophore mapping of compound **2** is shown in Fig. 6D.

In case of compound **3** also, Met 485 and Cys 525 helicase site residues of the enzyme were observed in polar interactions with the compound (Fig. 6E). From the protease site, the residues, His 57, Asp 81 and Ser 139 are involved in different polar interactions with the compound. His 57 made two interactions, a hydrogen bonding and an arene-hydrogen interaction with the oxygen of morpholine group and pyridine respectively. Ser 139 was also linked to oxygen of morpholine group through hydrogen bonding. Asp 81 made two polar interactions with imidazopyridine moiety and methylene group. The pharmacophore mapping of compound **3** is shown in Fig. 6F.

**Figure 7:**
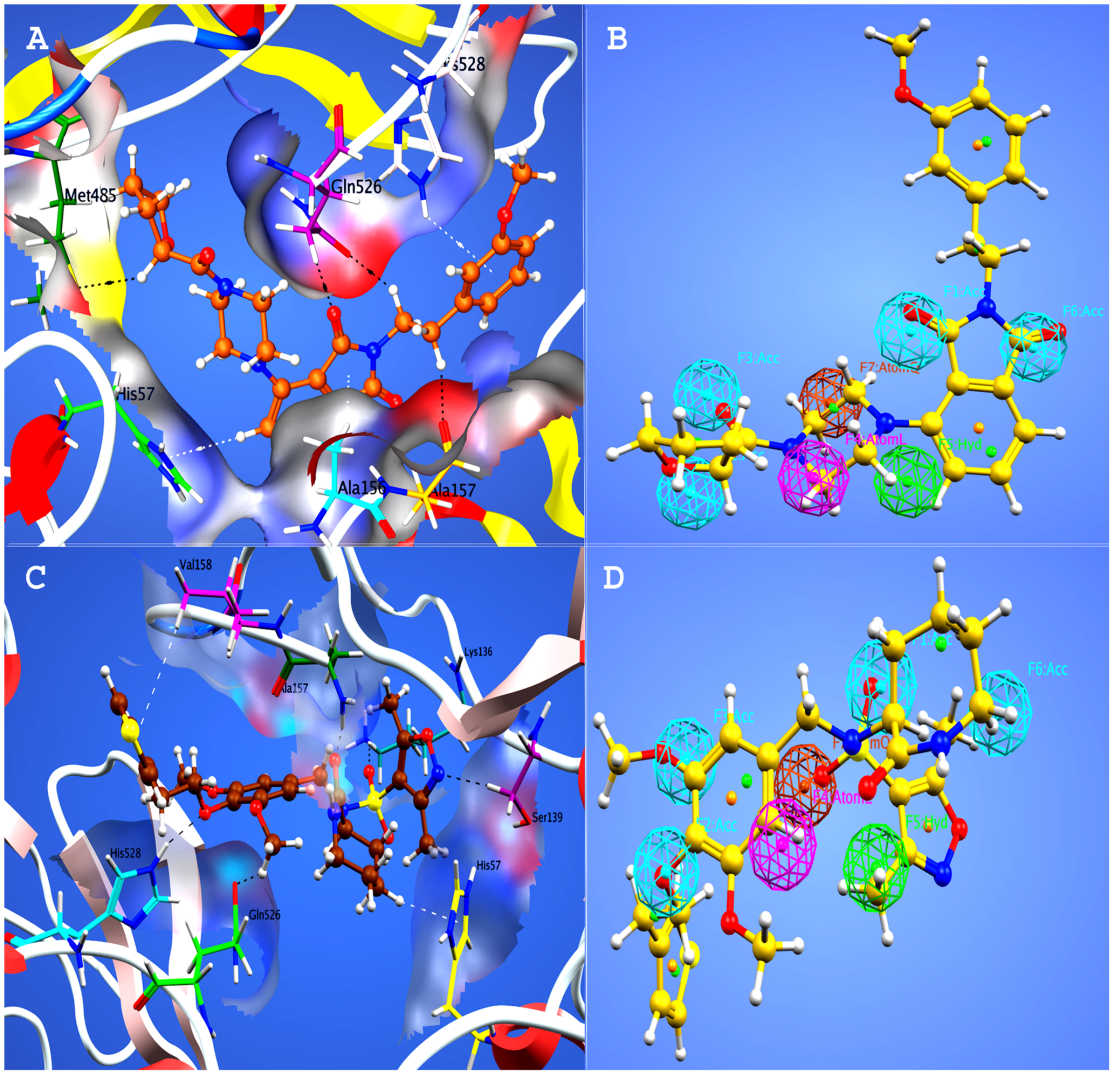
Docking conformations and pharmacophore mapping of compounds 13 and 14. (A) Three-dimensional representation of the interactions of compound 13 and target protein. (B) Pharmacophore mapping of hit compound 13. (C) Three-dimensional representation of the interactions of compound 14 and target protein. (D) Pharmacophore mapping of hit compound 14.

According to the docking score, comparatively low ranked hits also showed significant interactions with important residues of protease and helicase sites. For example, in compound **13 **it was observed (Fig. 7A) that isoindolinedione ring of compound form arene-hydrogen bonds to His57 and Ala 156 of protease site residues. Beside these residues of protease site Ala 157 also showed hydrophobic interaction to a methylene group of compound. Similarly, helicase site residues, Met 485, Gln 526 and His 528 were observed to be involved in intermolecular interactions with various groups of compound **13**. Met 485 and Gln 526 are involved in hydrogen bonding with furan group and carbonyl oxygen of isoindolinedione ring respectively. Gln 526 also showed a second polar hydrogen acceptor bonding to hydrogen of adjacent methylene group. An arene-hydrogen bonding was also observed between His 528 and phenyl ring of compound **13**. The pharmacophore mapping of compound **13** is shown in Fig. 7B.

A number of significant interactions were also observed with both protease and helicase site residues in case of compound **14** (Fig. 7C). His 57, Lys 136, Ser 139, Ala 157 and Val 158 from protease site were observed to be involved in interactions with the compound. His 57 made an arene-hydrogen interaction with azepan-2-one moiety of compound. Ser 139 was involved in a hydrogen donor interaction with nitrogen of isoxazole ring. Lys 136 formed a hydrogen donor interaction with the oxygen of sulfone group. Ala 157 was observed to be involved in bonding with the oxygen of azepan-2-one moiety of compound. Val 158 made an arene-hydrogen interaction with thiophene moiety of compound. Similarly Gln 526 and His 528 of helicase site residues were also involved in interactions with the oxygen atoms bonded to benzene ring. From the docking conformations of the selected compounds, it was observed that all the selected compounds are able to adopt suitable orientations within the binding pocket of HCV NS3/4A protease with some specific functional groups that interact to the important residues of the enzyme. The pharmacophore mapping of compound **14** is shown in Fig. 7D.

## Conclusion

In this study, the development of a chemical feature based 3D pharmacophore model of HCV NS3/4A protease inhibitors have been described from the crystal structure of NS3/4A protease in complex with a macrocyclic inhibitor interacting with both protease and helicase sites residues (4a92) via MOE pharmacophore constructing tool. The developed pharmacophore hypothesis consisted of seven key features including four hydrogen bond acceptors (Acc), one hydrophobic (Hyd), one for lone pair or active hydrogen (Atom L) and a heavy atom feature (Atom Q). The generated pharmacophore model was validated by a test database of seventy known inhibitors containing 55 active and 15 inactive/least active compounds. The validated pharmacophore model was used as 3D query in database screening. As a result of screening 1009 hits were retrieved and were subjected to filtering by Lipinski’s rule of five as a result of which 786 hits were selected for further assessment using molecular docking studies. The molecular docking result was analyzed for all the selected compounds on the basis of docking score, protein-ligands interactions, binding affinity predictions and binding energy calculations. Finally, 15 hits of different scaffolds and interactions with important amino acid residues of the active site were taken as lead candidates. These hits can be used as such and on further optimization as potential leads in developing novel inhibitors of HCV NS3/4A protease. These candidates having unique scaffolds have a strong likelihood to act as further starting points in the development of novel and potent NS3/4A protease inhibitors.
